# Semiconductor manufacturing wastewater challenges and the potential solutions via printed electronics

**DOI:** 10.1016/j.isci.2025.113576

**Published:** 2025-09-15

**Authors:** Sofia Sandhu, Ayoub Zumeit, Zhenyu Tian, Vincenzo Vinciguerra, Ravinder Dahiya

**Affiliations:** 1 Bendable Electronics and Sustainable Technologies (BEST) Group, Northeastern University, Boston, MA 02115, USA; 2 Barnett Institute, Northeastern University, Boston, MA 02115, USA; 3 STMicroelectronics, Catania, Italy

**Keywords:** chemistry, engineering, materials science

## Abstract

The rapid expansion of the semiconductor industry has not only accelerated global digital transformation but also brought serious environmental concerns, particularly through the discharge of hazardous, toxic, and persistent wastewater. Traditional semiconductor manufacturing is highly water and energy intensive, relying on critical and often toxic materials—many with proprietary or unknown chemical compositions. This review outlines the environmental impact of semiconductor wastewater, highlighting key contaminants and chemicals used in the process. Due to the complexity and sometimes the opacity of wastewater composition, current treatment technologies face significant limitations. The article also explores the potential for reducing environmental harm by transitioning toward minimal liquid discharge systems. In particular, it highlights alternative technologies such as printed electronics, which offer a promising path forward by minimizing the use of toxic chemicals, reducing energy consumption, and significantly lowering wastewater output. These strategies represent a realistic and necessary shift toward more sustainable semiconductor manufacturing practices.

## Graphical abstract

**Figure:**
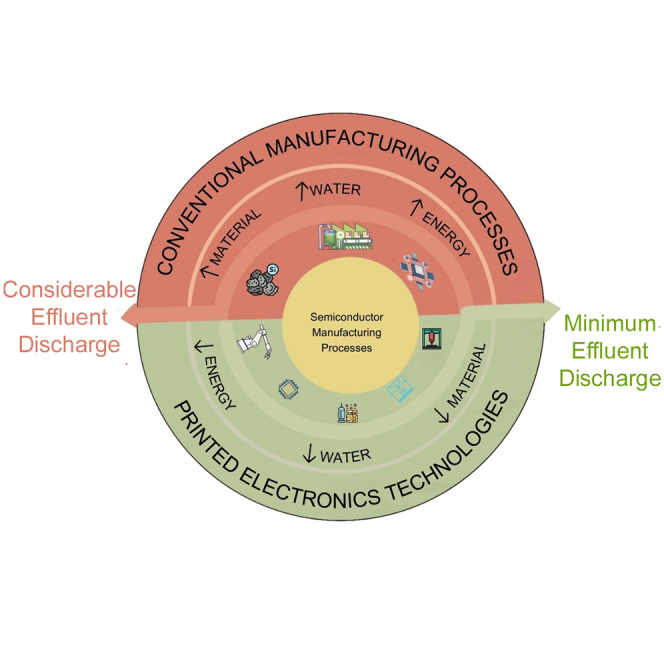

## Introduction

The evolution of semiconductor technology, since the days of vacuum tubes to today’s nanoscale transistors, has propelled technological innovations and advancements across myriad industries, thanks to the relentless quest for miniaturization that closely followed Moore’s law^1^^,^^2^ and fueled exponential gains in computing performance, enabling everything from supercomputers to smartphones and the widespread digitalization.^3^^,^^4^^,^^5^ The use of new materials is another pillar that is shaping the semiconductor industry, as may be noted from the recent emergence of transparent microLED-based televisions having screens that look like a sheet of glass when not in use but display incredibly high-definition images when needed, thanks to their high pixel density.

The growing digitalization of various traditional sectors of economy and the emerging new applications mean the demand for electronics will continue to grow and with it, the semiconductor manufacturing will grow too. In fact, the global market’s valuation of semiconductor industry anticipates a rise from the current USD 544.78 billion to USD 1,137.57 billion in the next 10 years.^6^ This rapid expansion of semiconductor industry will inevitably lead to greater ecological footprint as traditional electronics manufacturing heavily depends on highly resource-intensive processes such as lithography, which require considerable amount of energy, high-purity water, and other resources. Putting this in perspective, in 2021, high-purity water requirements of the semiconductor corporations ranged from 2.3 to 163.7 × 10^6^ m^3^ and energy consumption was in the range of 0.41–32.3 × 10^9^ kWh.^7^ In addition, these processes use a wide variety of toxic and hazardous chemicals that are eventually released into the wastewater stream and pose hidden risks to the environment and human health.

Considering the health and environmental risks associated with toxic wastewater release, various physical, chemical, and biological studies^8^ have been carried out during the last decade with the aim of optimizing the wastewater treatment efforts. However, semiconductor wastewater is a niche area that involves many unknown and hazardous contaminants as provided in Table 1 and requires wide-ranging knowledge and skills on the chemical and physical treatment of wastewater. Standard semiconductor fabrication is a complex process involving many steps including material synthesis, wafer manufacturing, oxidation, photolithography, ion implantation, etching, deposition of thin films, etc., all of which produce substantial waste including hazardous chemicals.^19^ Traditional treatment methods to remove or reduce these contaminants include physical and chemical methods such as adsorption, coagulation, flocculation, aggregation, precipitation, and membrane separation and advanced oxidation processes such as catalytic wet oxidation, electrochemical oxidation, etc.^20^^,^^21^^,^^22^ Traditional biological treatment includes algae-based biosorption processes and biological activated carbon reactors to name a few.^23^^,^^24^ These methods are often found to be insufficient to fully mitigate the environmental harm, and therefore, there is a need for further research and innovation in wastewater treatment processes, particularly to achieve zero liquid discharge (ZLD).^25^

**Table 1:** Key pollutants and their concentration in wastewater and environmental risks

Pollutants	Typical concentration	Environmental Risks	Notes	Reference
PFAS	Up to 78,000 ppt	Bioaccumulation, toxicity; linked to cancer, immune disorders, and ecological harm	Includes short-chain variants (C3/C4), highly persistent and mobile	Jacob et al.^9^; Jacob and Helbling^10^; Liu et al.^11^
COD	>70,000 mg/L	Depletes oxygen in water bodies, harming aquatic life	Low biodegradability (BOD/COD ≈0.124) due to recalcitrant organics	Noman et al.^23^; Wong et al.^12^; Sadafale and Gaikwad^13^
TS	∼4,500 mg/L	Clogs waterways, disrupts ecosystems, reduces light penetration	Includes fine oxide/silicon particles (size <0.45 μm)	Noman et al.^23^; Sadafale and Gaikwad^13^; Lin and Yang^14^
pH	Up to 9.5	Alters water chemistry, harms aquatic life, corrodes infrastructure	Caused by alkaline cleaning/etching agents	Noman et al.^23^; Sadafale and Gaikwad^13^; Lin and Yang^14^
Copper (Cu)	5–100 mg/L	Toxic to aquatic organisms, disrupts food chains	Common in CMP wastewater; exceeds regulatory limits	Wang et al.^15^; Lippert et al.^16^
Heavy metals (Zn, Ni, etc.)	Trace: tens of mg/L	Chronic toxicity, bioaccumulation in ecosystems	Varies by process (e.g., etching, plating)	Noman et al.^23^; Sadafale and Gaikwad^13^; Hsu et al.^17^
TDS	Can exceed 100,000 lbs/day (large fab)	–	Major concern for water reuse and discharge	Noman et al.^23^; Lin and Yang^14^
Recalcitrant organics (e.g., TMAH, azoles)	Present	–	Poorly biodegradable, hard to detect and treat	Sadafale and Gaikwad^13^; Cheng et al.^18^
Fine particulates	SS < 0.2 mg/L	Smothers aquatic habitats, reduces biodiversity	Particles too small for standard filtration	Noman et al.^23^; Lin and Yang^14^

BOD, biochemical oxygen demand; COD, chemical oxygen demand; SS, suspended solids; TDS, total dissolved solids; TS, total solids

Thus, environmental implications, extending far beyond the excessive consumption of water and energy, underline the need for maintaining the toxic contaminants released into the wastewater stream to non-harmful levels and strict regulations. This could be achieved either by treating the wastewater or through the development of alternative manufacturing processes that do not require hazardous chemicals. In this regard, new manufacturing routes based on additive manufacturing and printed electronics technologies are noteworthy. Printed electronics has much lower ecological footprint when compared with conventional silicon (Si)-based fabrication processes as it allows development of similar devices with lesser number of processing steps, lesser material, minimal material wastage, lower reliance on hazardous chemicals, and reduced use of resources such as energy, water, and materials.^26^^,^^27^^,^^28^^,^^29^^,^^30^^,^^31^ Further, printed electronics can offer several advantages by allowing electronics in flexible form factor, cost-effectiveness, scalability, and the ability to offer customized solutions to adapt to existing infrastructures.^32^^,^^33^^,^^34^ Printed electronics has been successfully used for the development of various types of devices such as radio identification tags, bio/chemical/physical/optical sensors, energy harvesting and storage devices, memristors, transistors, etc.,^35^^,^^36^^,^^37^^,^^38^^,^^39^^,^^40^^,^^41^^,^^42^ and it is also attractive in terms of recycling and reuse of materials and overall circularity in semiconductor manufacturing.^43^^,^^44^^,^^45^^,^^46^^,^^47^^,^^48^^,^^49^ For example, the sludges generated from wastewater treatment could be used to develop substrates or inks for printed electronics. However, the current printed electronics technology is somewhat distant from producing active devices (e.g., transistors) and circuits with performances at par with conventional silicon-based electronics.^50^^,^^51^^,^^52^ Nonetheless, recent advances in additive manufacturing allowing printing of metals, insulators, semiconductors, and materials for packaging^47^ offer an attractive alternative for the development of electronics with negligible material waste and wastewater contaminants.

**Figure 1:**
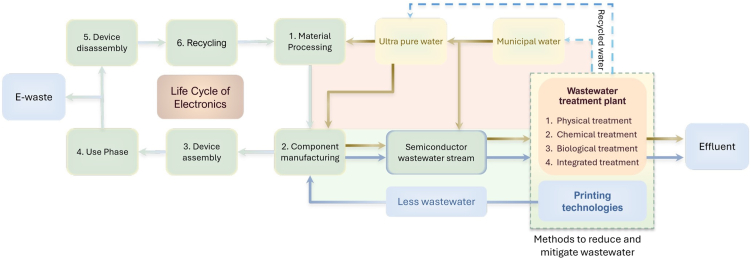
Overview of the end-of-life cycle of electronics and the water cycle during the semiconductor manufacturing processes from water usage to wastewater release to recycling

This review article discusses the above-mentioned complexities and solutions for the contaminated wastewater generated by semiconductor manufacturing, as well as the trade-offs for alternative pathways to achieve zero contaminant discharge/ZLD during chip fabrication. Figure 1 provides a visual overview of the life cycle of electronics from materials processing to recycling and focuses on the water cycle (from water consumption to effluent release to recycling) during the manufacturing process. The article starts with the need for ultrapure water (UPW) in semiconductor manufacturing, various ways for generation of UPW, and volume required for the manufacturing processes. This is followed by a discussion on various traditional steps for chip fabrication, which leads to the release of toxic contaminants in wastewater. Following this, we discuss various approaches for the treatment of wastewater, particularly the ones that allow the reuse of water in semiconductor manufacturing, and existing regulations and policies to control the wastewater. We then present the viability of printed electronics as a solution for current challenges related to wastewater contaminant from semiconductor manufacturing. To this end, conventional fabrication steps for the development of a transistor (i.e., basic building block for integrated circuits [ICs]) are compared with methods such as hybrid fabrication, i.e., mix of traditional fabrication and printed electronics, and fully printed electronics used for the development of similar device. The article concludes with the outlook for the future.

## Water consumption and wastewater generation in semiconductor industry

### Need for UPW

Semiconductor manufacturing processes are highly intricate and resource intensive. The production of semiconductors requires UPW, which is regarded as an industrial solvent and holds importance equivalent to silicon—the workhorse material for semiconductor industry. UPW is water that has been highly purified, removing all minerals, particles, bacteria, microbes, and dissolved gases. It is also known as de-ionized water, in that all ion components have been removed. UPW is the ultimate cleaning agent, necessary during multiple steps in a device fabrication process. These include repeated steps for wafer cleaning, rinsing and surface conditioning, chemical mechanical planarization, wet etch, etc. Typically, the semiconductors involve ultrafine nanoscale processes, and even the tiniest speck remaining after each process can cause critical errors. The use of UPW to cleanse the wafers between each step ensures the highest standards of cleanliness are maintained and semiconductor productivity (yield) is maximized. Thus, UPW plays a key role in the production of flawless semiconductors.

The semiconductor processes demand the highest level of purity—at least 18 MΩ cm (at 25 °C) of electrical resistivity is required with extremely low levels of pollutants.^53^^,^^54^ The generation of UPW requires high energy and results in substantial volumes of wastewater, which needs to be treated timely and effectively. For example, typical fabs can use up to 30–50 MW of peak electrical capacity, which is enough to power a small city.^55^ Further, to make 1,000 gallons of UPW, approximately 1,600 gallons of municipal water are needed.^56^ The fabrication of ICs on a 300-mm wafer can require approximately 2,200 gallons of water, including 1,500 gallons of UPW.^56^ This means a large fabrication facility that processes about 40,000 wafers a month can use up to 4.8 million gallons of water per day. This equates to the annual water consumption of a city of 60,000 people.^7^

Water covers 70% of our planet, and it is easy to think that it is plentiful. However, freshwater—the stuff we drink, bathe in, and irrigate our farm fields with—is incredibly rare. Only 3% of the world’s water is fresh water, and two-thirds of that is tucked away in frozen glaciers or is otherwise unavailable for our use. Further, availability of water is also impacted by global warming.^57^ Consequently, the large ecological footprint of the semiconductor industry can also impact the sustainable supply of water.^58^ In addition to UPW, the semiconductor industry also requires freshwater for the functioning of various servers.^59^ With the rise generative AI products, cooling of servers is gaining more importance as water usage for AI servers is anticipated to hit 6.6 billion m^3^ by 2027.^60^ Clearly, the semiconductor industry needs to adopt sustainable water practices to maintain its growth and competitiveness. Some large companies have already implemented water recycling systems. For example, Taiwan Semiconductor Manufacturing Company (TSMC)’s 2023 sustainability report mentions it reclaimed 286.35 million metric water.^61^ TSMC has been investing in reclaimed water technology since 2015.^62^ To achieve higher recycling rate, it is important to treat the wastewater released during semiconductor manufacturing processes, and, for this, it is important to understand the nature and amount of contaminants in the wastewater released from these processes.

### Wastewater generation

The conventional semiconductor fabrication processes typically involve steps such as material synthesis, wafer manufacturing, oxidation, photolithography, etching, deposition and ion implantation, metallization, chemical mechanical polishing (CMP), electrical wafer sorting, electrical die sorting (EDS), and packaging.^63^ The semiconductor manufacturing heavily relies on photolithography, which leads to subtractive processes. The manufacturing of semiconductors involves more than 400 chemical products, and the IC fabrication process as a whole generates substantial volumes of wastewater containing a wide array of hazardous chemical pollutants including heavy metals, acids, alkalis, solvents, and other toxic substances.^23^ It is estimated that semiconductor wastewater accounts for approximately 28% of the total untreated industrial wastewater discharged into the environment, posing significant environmental and human health risks.^64^

**Figure 2:**
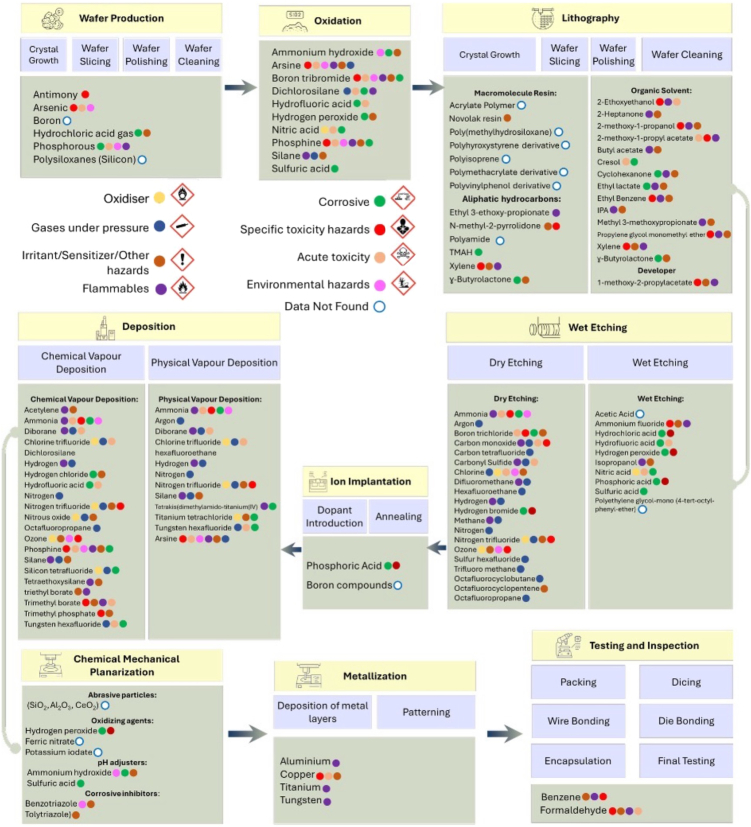
Conventional manufacturing processes in a semiconductor industry Various fabrication steps typically adopted in the conventional semiconductor industry and the chemicals commonly used in each process along with their potential hazards.

It is to be noted that the information related to chemicals used in semiconductor fabrication is often generalized in the literature and not based on actual data. Figure 2 provides an overview of the key fabrication processes and the associated chemicals they use. The detailed data related to the quantity and volume of chemicals employed in semiconductor industry are not available in the public domain.

Si is the primary material that is used in wafer production. During the wafer manufacturing process, high-purity Si is extracted from silicon dioxide (SiO_2_) by using hydrochloric acid (HCl) gas, which leads to the formation of trichlorosilane, and this, when decomposed at high temperature, deposits pure Si. Gallium arsenide (GaAs) is another dominant material widely used in opto-electronic applications. Lapping and polishing of wafers based on these materials is typically needed to achieve a smooth surface and enhance their overall quality, and this process involves chemicals such as aluminum oxide slurry or silicon carbide abrasive particles. Post-polishing, cleaning of the wafers is carried out using cleaning agents to remove organic contaminants, metallic contaminants, and organic residues. Some of the common cleaning agents are acids (hydrochloric acid [HCl], sulfuric acid [H_2_SO_4_]), bases (ammonium hydroxide [NH_4_OH], ethoxylated amine), and oxidizing agents (hydrogen peroxide [H_2_O_2_], 5% sodium hypochlorite [NaOCl]).^65^ Wafer fabrication potentially has the highest environmental impact on the entire semiconductor value chain since it involves the management of huge quantities of raw material, energy, waste, and emissions.

In the oxidation process, a thin layer of dielectric (e.g., SiO_2_) is formed on the wafer by exposing the wafer to oxygen or steam at high temperatures. There are a variety of oxidation methods, such as thermal oxidation, electrochemical anodic oxidation, and plasma-enhanced chemical vapor deposition (CVD). Among them, the thermal oxidation procedure, performed at a high temperature, is most widely used. Thermal oxidation can be either wet or dry depending on the desired thickness. Dry oxidation only uses oxygen, whereas wet oxidation uses both oxygen and vapors. To eliminate impurities from SiO_2_ surface, a series of washing/cleaning cycles are needed, including standard clean 1 (mixture of NH_4_OH, H_2_O_2_, and H_2_O) followed by standard clean 2 (mixture of HCl, H_2_O_2_, and H_2_O) and hydrofluoric acid (HF).

Oxidation is followed by photolithography, which involves transferring a pattern onto the wafer using a light-sensitive photoresist. During this process, macromolecule resins (xylene, Novolak resin, aromatic compounds, and various organic solvents), photoresist developer (a strong base such as tetramethylammonium hydroxide [TMAH], a mild base such as N-methylpyrrolidone [NMP]), and other chemicals are utilized. Further, organosilicon compounds such as hexamethyldisilazane are used as adhesion promoters to enhance the bond between the wafer and the photoresist. Additionally, the use of per- and polyfluoroalkyl substances (PFAS), a group of synthetic chemicals during the photolithographic process, is quite prevalent.^63^ These substances, known as “forever chemicals,” are extremely difficult to degrade and quite persistent in the environment and have the potential for bioaccumulation.

The etching process is another critical step in semiconductor fabrication. In this process, a pattern is transferred by lithography to the layer to be etched or on a hard mask. Then the exposed area is etched away, while the remaining area remains intact. The two fundamental types of etchants are liquid phase (or wet etching) and plasma phase (or dry etching). The choice of method to be implemented depends on the type of material to be etched. Dry and wet etching processes involve use of many gaseous compounds and acids (CH_3_COOH, HF, HNO_3_ etc.), ammonium fluoride (NH_4_F), ammonium hydroxide (NH_4_OH), etc., followed by removal of the photoresist using H_2_SO_4_.^66^

There are two types of deposition processes, viz., CVD and physical vapor deposition, and both processes involve various types of gaseous compounds as shown in Figure 2. CMP involves chemicals such as a slurry with abrasive particles and chemicals that facilitate the material removal. The process further requires a large amount of UPW (around 40%) to remove the waste slurry and small particles from the wafer surface and as a result, produces approximately an equal amount of wastewater. Generally, the CMP wastewater consists of 5%–10% of nano-sized particles (such as SiO_2_, CeO_2_, or Al_2_O_3_), oxidizing agents (such as H_2_O_2_, Fe(NO_3_)_3_, CuSO_4_, KIO_3_, etc.), pH buffers (such as KOH, NH_4_OH, etc.), and surfactants.^67^

Metallization is another key fabrication step needed to connect various circuit layers on the wafers. Typically, metals such as aluminum (Al), copper (Cu), titanium (Ti), and tungsten (W) are used in ICs. During packaging process, semiconductor products are assembled using benzene (C_6_H_6_) or formaldehyde (CH_2_O).^68^ Finally, EDS begins with electrical testing to check if the chips meet the required quality level. There are five stages of EDS process: (1) electrical test and wafer burn-in, effectively diminishing defects at the initial stage of production; (2) pre-laser (hot/cold), to determine whether each chip on the wafer is functional or faulty; (3) laser repair and post-laser, to repair the faulty chips using laser; (4) tape laminate and back grinding, to assemble thin products, such as IC cards that are used in transit cards or passports; and (5) inking, to distinguish defective chips by special ink marks. During testing and inspection, benzene and formaldehyde are mainly used. Another chemical that is ubiquitously used in each process discussed above is isopropyl alcohol.

Depending on the chemical nature of the chemical used in each process, the wastewater discharges are categorized into four waste streams at the final stage: (1) HF acid wastewater; (2) acidic, caustic, and organic wastewater; (3) CMP wastewater; and (4) high-concentration liquid waste. Regarding the quantity and composition of chemicals used, the data from two factories (A and B) of a leading company show that the chemicals used in photolithography were 21% (A) and 26% (B) of all chemical products and more than 97% among them were chemicals containing trade secret ingredients.^69^ More than 150 pure chemical substances were used in about 430 chemical products in a semiconductor company; about 40% of these chemical products contained trade secret ingredients. In photolithography, one of the most widely used processes in semiconductor manufacturing, nearly all products (about 98%) contained trade secret ingredients, with an average number of approximately two per product. The amount of chemicals used was 46,850 and 45,628 tons in factories A and B, respectively. The large amount of trade secret ingredients used in semiconductor manufacturing makes it difficult to obtain accurate composition of the chemicals.^70^ Nonetheless, the studies show that the wastewater streams contain perfluorooctanoic acid (0.34–3.35 mM), TMAH (5–60 g/L), CaF_2_ (700–800 mg/L), KOH (50–1,000 mg/L), NH_3_–N (2.8–3.9 mg/L), turbidity (nephelometric turbidity unit (NTU)) (1,044–6,390), pH (6.3–9.8), 0.1–5.3 g/L of total solid, 0–0.2 mg/L of suspended solid, COD (175–5,000 mg/L), sulfates (45.6–58.2 mg/L), etc.^17^ Further, the organic solvent particles range from nano- to micro-sized, which is considered as the main pollutant. Varying composition and characteristics of these waste streams is what poses difficulty in treating them.

### Regulations and policies

Several regulations have appeared from time to time to control wastewater contamination from large-scale semiconductor manufacturing. For example, in the United States, the Clean Water Act regulates the release of pollutants in surface waters^71^ and the US Environmental Protection Agency has also released a guide in this regard.^72^ Specifically, pollutants are regulated by the Maximum Achievable Control Technology index. Likewise, the European Environment Agency observes the effectiveness of the EU Policies on the environmental performances of European industry. Specifically, the industrial pollutants are regulated in the European Union by directives such as the Industrial Emissions Directive, the Medium Combustion Plant Directive, the Eco-design Framework Directive, European Union’s Emissions Trading System, the Water Framework Directive, and the Urban Wastewater Treatment Directive.^73^ The companies involved in the modern semiconductor sector generally follow these regulations and regularly report the use of the resources and on the environmental impact of their production lines. For example, STMicroelectronics constantly assesses and monitors the resources and summarizes the same in its sustainability report.^74^ The whole water cycle in a production plant is monitored to ensure the UPW entering the plant can also exit in the same condition. However, it is also to be noted that most of the semiconductor production plants are nowadays based in the East. Considering this, the issue of wastewater release from semiconductor industry appears to be an “out of sight, out of mind” topic for the developed countries in the West. Undoubtedly, the leading semiconductor manufacturing companies in the East may be operating their own semiconductor wastewater treatment plants. However, the gaps between prevailing regulations and national standards in different regions may allow dilution of the measures needed to reduce chemical hazards in semiconductor wastewater.^75^ This is compounded by the diversity of chemical substances used in conventional fabrication, which makes it difficult to understand what substances are exactly used. The “unknown” or “unrecognizable” chemicals complicate the efforts to assess and mitigate the environmental and health risks associated with semiconductor manufacturing and pose hurdles for designing suitable wastewater treatment processes.^69^ Obtaining detailed hazard and chemical composition information is a daunting task due to trade secrets, patents, and the proprietary nature of many chemicals. Without a comprehensive understanding of the chemical used in the industry, it is extremely difficult to determine what types of pollutants need to be tested and hence what wastewater treatment technologies need to be designed. On top, new chemicals are being introduced to industrial operations at a much faster pace than the health and environmental studies needed to understand the risks.

### Semiconductor water recycling and wastewater treatment technologies

Over the past 8 years, waste generation in the semiconductor industry has nearly doubled.^76^ This waste includes chemical waste, solid waste, wastewater, slurries, abrasives, and packaging waste. Hazardous waste, such as unused chemicals containing PFAS and waste slurries from processes such as CMP, poses environmental and human health risks if not properly treated. Water management strategies vary based on the location of fab and differences in water sources and recycling rates. Fabs in regions with high water scarcity, such as Taiwan, emphasize water reuse and recycling, achieving rates of around 80%, while those in Europe typically have lower recycling rates (10%–14%).^77^ However, climate change is also forcing policy makers to increase recycling of water everywhere. While recycling rates for reuse in other industries are high (around 70%), only a small percentage can be reused for semiconductor manufacturing.

Based on the complex nature of waste streams, appropriate treatments are required.^23^ The heavy lifting for municipal wastewater is carried out by the biological process (secondary treatment with aerobic sludge). Most of the organics are removed at this stage by microbes. Traditional chemical treatments, including coagulation, flocculation, and advanced oxidation processes, are often employed but are not entirely effective in removing all contaminants. Because of the special compositions of semiconductor wastewater (e.g., strong acids and high chemical oxygen demand), more pre-treatment steps are necessary before the biological process of wastewater treatment to lower the toxicity.^78^^,^^79^ Without specifically designed treatment technologies, persistent toxic substances in semiconductor wastewater may still be discharged into water bodies, adversely affecting aquatic ecosystems and human health. For example, PFAS contamination in semiconductor wastewater is a growing issue, as these substances are resistant to degradation and have hydrolytic lives of more than 41 years. In Japanese rivers and Taiwan river waters, PFAS concentration was found to be in the concentration range <5.2 to 10 ng/L and 82–5,440 ng/L, respectively, which is higher than its accepted safe limit of 4 ng/L. The potential health risks associated with PFAS include developmental issues, immune system disorders, an increased risk of certain cancers, etc., all of which underscore the urgency of addressing this problem.^80^ It is to be noted that several studies have been carried out globally on the removal of fluorine compounds (e.g., polyfluorinated alkyl substances or fluoroquinolones) in wastewater, but the research on fluoride removal in semiconductor wastewater has not been carried out as intensively as needed. The result of removing fluorine compounds from semiconductor wastewater is CaF_2_, known as fluorspar, and in this direction, Singapore ESGL has developed a circular economy solution for recycling sludges containing fluorspar produced by the semiconductor industry.^81^

Several studies have been conducted on pilot scale to treat the wastewater from semiconductor industries, but they fail to present a complete picture due to proprietary concerns.^82^^,^^83^ Even with the known chemicals, the complexity surrounding their composition makes wastewater difficult to treat. Further, the industry’s reluctance to disclose detailed information about the chemicals used, due to proprietary concerns, hampers efforts to develop effective wastewater treatment solutions. For instance, more than 97% of chemicals used in the photolithography process contain trade secret ingredients, with the average number of trade secret ingredients per product being 2.3, whose toxicity and chemical nature are unknown.^69^ Handling of semiconductor sludge remains a challenge for the zero-discharge goal because of the lack of efficient technologies for recycling these wastes. In addition, semiconductor wastewater handling is challenging due to the complexity of treatment procedure and greater toxicity. Thus, as fabrication process becomes complex, there is a need to understand the intricate semiconductor wastewater treatment process.

Based on the above discussion, it appears that (1) more progressive types of processes need to be developed to treat the wastewater released from the semiconductor industry and detailed information related to chemicals used is needed to attain the goal of zero discharge and (2) alternative manufacturing routes, which have the potential to reduce the usage of water and hence release of effluents, need to be explored.

## Printed electronics as a route to achieve minimal liquid discharge

It is clear from the above discussion that the treatment of wastewater streams alone is not enough and to achieve zero-contaminated wastewater discharge there is a need to look beyond the conventional techniques. In this regard, emerging technologies such as printed electronics could offer solutions to mitigate the ecological footprint of the semiconductor manufacturing.

Conventional semiconductor manufacturing, particularly the silicon-based complementary metal oxide semiconductor (CMOS) technology, involves numerous processing steps, such as vacuum deposition, lithography, high-temperature diffusion, electroplating, and etching, which collectively contribute to significant wastewater discharge. Processes such as lithography, which involves coating the wafer with photosensitive materials called photoresist to transfer a pattern through a mask, are highly energy intensive and rely on hazardous chemicals and generate considerable volumes of toxic wastewater and chemical waste. The manufacturing of modern semiconductor devices, such as those utilizing the CMOS technology, involves hundreds of distinct process steps.^84^ High-performance CMOS logic circuits share many fabrication steps with other silicon-based devices, including flat panel displays and photovoltaics, as well as other applications that utilize nano- or micro-fabrication processes.^85^ The fabrication of these electronic devices fundamentally involves the construction of a rectangular “die,” which consists of a set of patterned layers of doped silicon, insulators/dielectrics, and metals that form transistors, which are the main building blocks of any digital microchip.^84^ For example, the number of transistors in a modern 32-MB microchip can start from hundreds of millions, depending on the type of memory and specific design considerations.^86^^,^^87^ Other processes in semiconductor manufacturing include deposition of thin films on the wafer using physical or chemical methods such as CVD and oxidation and selective etching to transfer the pattern onto the films.^47^ In addition, processes related to the introduction and control of dopants into transistor active regions by ion implantation are critical. These high-energy processes contribute significantly to wastewater generation.^85^

**Figure 3:**
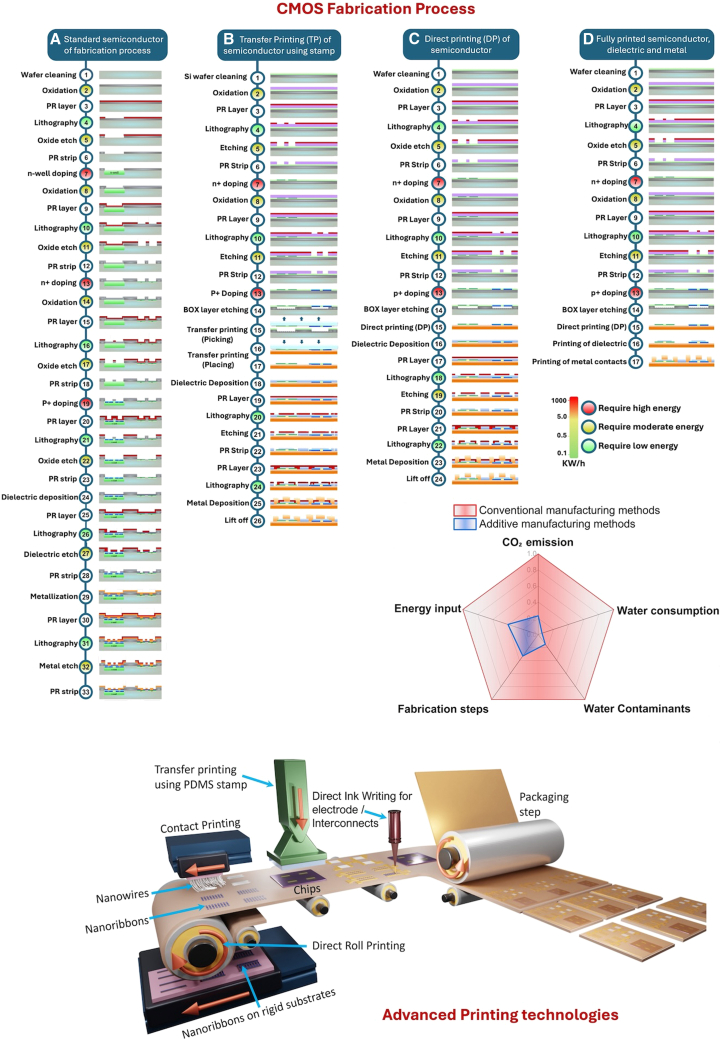
Qualitative comparison of fabrication steps in conventional semiconductor fabrication versus various emerging printing electronics technologies Conventional semiconductor fabrication with emerging printing electronics technologies (A) such as transfer printing of semiconductor layer only (B), direct printing of semiconductor layer only (C), and full printing (D), that is, printing semiconductor, dielectric, and metal. The number of fabrication steps decrease from left to right, i.e., from energy-intensive conventional method (leftmost) to fully printed electronics (rightmost), highlighting the reduction of energy use and environmental impact.

Figure 3 provides a qualitative overview of the key fabrication steps in standard semiconductor fabrication. The steps such as defining high-mobility n-type and p-type semiconductors for low-power logic circuits, low-leakage and high-k gate dielectrics for transistors, and high-conductivity metals for electrodes and interconnections are all essential to achieving the desired device characteristics.^88^ To achieve this, wafers are processed through numerous cycles, which can range from tens to hundreds of cycles of cleaning, photoresist coating, lithographic exposure, development, oxide deposition, etching, and photoresist removal, with each step contributing to the overall complexity of the process. These repetitive processing steps, particularly during lithographic procedures and etching, produce significant amounts of acid-base waste.^89^ For instance, etching metal films to form transistor contacts generates considerable acid-base waste, especially during microchip fabrication where the source, drain, and gate are implanted with metal contacts. This process often requires the repetition of metallization and etching steps multiple times to establish the necessary interconnects and interlayer connections (vias).^90^

As shown in Figure 3, transitioning from standard semiconductor fabrication to fully printed technologies demonstrates a clear reduction in the number of steps, material usage, and associated material wastage, leading to more environment-friendly manufacturing processes. Printing technologies also offer significant potential in reducing the energy requirements and chemical waste associated with traditional semiconductor fabrication methods.^91^ Processes such as oxidation and ion implantation, which are essential for creating the desired electrical characteristics in CMOS-based devices, typically operate at temperatures reaching up to 1,200°C.^92^ Doping involves implanting ions into the silicon substrate, followed by annealing at high temperatures to drive the dopants into the desired profiles. This annealing process is energy intensive, as maintaining a furnace at high temperatures (800°C–1,200°C) requires substantial power.^93^ It is difficult to provide an accurate estimate of electricity consumption for global ion implantation, as the data related to actual energy are not readily available. Further, the energy used could vary significantly depending on the specific parameters of the ion implanter, the implant dose, the desired implantation depth, and the overall system efficiency. Similarly, if we consider the case of wafer thermal oxidation, where the silicon on the wafer is made to react with oxygen or N_2_O at temperatures between 800°C and 1,100°C to form the high-quality silicon dioxide, the energy consumption during this process can widely vary depending on factors such as the specific equipment used, the scale of the operation, the desired oxidation thickness, etc. For instance, depositing a 100-nm oxide layer at a typical deposition rate of ∼1 nm per minute would take approximately 100 min. The energy needed by a furnace operating at 500 V and 20 A, can be calculated as follows: Energy (kWh) = voltage (V) × current (A) × time (h)/1,000. This equates to 500 V × 20 A × (100/60) h/1,000 = 16.67 kWh. Semiconductor fabs typically process wafers in batches, with a standard batch comprising 25 wafers. Scaling up to thousands of wafers results in significant energy consumption and a substantial carbon footprint, especially if powered by non-renewable energy sources.

In contrast, advanced printing technologies bypass some of these high-energy processing steps, thereby reducing both energy consumption and wastewater generation.^29^ As illustrated in Figures 3C and 3D, these new technologies involve the transfer of pre-fabricated micro- or nanostructures onto flexible substrates through transfer printing and direct roll printing (DRP) methods.^28^^,^^52^^,^^94^^,^^95^ These techniques are specifically mentioned here as they enable the fabrication of silicon-based flexible transistors at lower temperatures with lesser lithography steps and lesser chemically intensive processes, thus providing a fair comparison with silicon-based standard fabrication technique presented in Figure 3A. Other printing technologies such as offset printing, gravure printing, screen printing, etc., which are typically used for high-volume printing, are not included in this comparison as they are normally not used for fabrication of transistors.^96^^,^^97^^,^^98^ These techniques may also introduce some contamination in the form of volatile organic compounds from inks and pastes, which may contain pigments based on heavy metals such as lead, chromium and cadmium. Nonetheless, the contaminants and wastewater generation are still far below the requirements for standard micro/nanotechnology.

Figure 3B highlights how transfer printing, demonstrated in Figure 3B, utilizes elastomeric stamps—typically made from polydimethylsiloxane—to transfer semiconducting nanostructures from a donor to the receiving substrate. While this method has shown significant promise in fabricating flexible electronics, the residues left over from viscoelastic stamps are difficult to be removed.^99^ Additional chemical etching steps needed to remove the residues lead to toxic elements in the wastewater and hence dilutes the benefits of transfer printing method. The micro/nanopatterns needed on these transfer stamps also require extra processing steps, often involving lithography and etching, which contribute to acid-base waste. Achieving high printing yield, precise registration, and reproducibility are some other challenges associated with this method. To address these limitations, alternative transfer printing schemes such as DRP have been developed. DRP offers a robust approach for transferring inorganic semiconducting nanostructures onto flexible substrates without the need for intermediate stamps, which leads to reduced processing steps such as photolithography and lower chemical usage, as shown in Figure 3C.^37^^,^^52^ Advanced printing technologies that allow fully printed devices, by combining DRP of inorganic semiconductors with direct printing of functional inks containing dielectrics, and metals, as shown in Figure 3D, offer a pathway to further reduce the number of fabrication steps, including lithography and etching. This approach minimizes material wastage and reduces wastewater production while promoting resource-efficient and sustainable electronics. Further, this process allows room temperature printing of a complete transistor structure on a flexible substrate, thereby avoiding energy-intensive stages such as oxidation, doping, and annealing. Furthermore, the absence of photolithography and chemical etching steps means no hazardous chemicals are required.

It is clear from the above discussion that printed electronics and additive manufacturing technologies offer considerable environmental advantages over conventional semiconductors as compared in Table 2 and printed circuit board (PCB) fabrication by utilizing low-toxicity, often water-based inks, thereby significantly reducing chemical effluents and the overall ecological footprint of device manufacturing.^5^^,^^100^ In this regard, life cycle assessment (LCA) studies report that additive methods can reduce the carbon footprint by ∼75%, with CO_2_ emissions decreasing from ∼104 kg CO_2_-eq/m^2^ for traditional boards to ∼24.5 kg CO_2_-eq/m^2^ for additively manufactured equivalents.^101^ Water consumption is also significantly lowered by up to 95% as additive approaches typically require only 10–100 L/m^2^, compared to ∼1,500 L/m^2^ in conventional processes, which involve extensive rinsing and wet chemical treatments.^102^ Furthermore, hazardous chemical emissions are reduced by around 85%, due to the elimination of most toxic etching, development, and plating steps that dominate the environmental impact in traditional PCB fabrication.^103^ These findings support the vision of near-zero discharge sustainable electronics manufacturing.^100^^,^^104^^,^^105^^,^^106^ Conventional silicon chip fabrication and PCB etching are highly resource and energy intensive, relying on many subtractive lithography steps with large chemical and water inputs, whereas additive printing deposits material only where needed without complex lithography or etching.^106^^,^^107^ This results in much lower energy demand in manufacturing.^100^ For example, printed organic photovoltaic devices have been shown to achieve 50%–60% shorter energy payback times than comparable amorphous-silicon solar cells, indicating a substantially reduced manufacturing energy footprint.^108^ An LCA of consumer devices found that adopting additive printed circuit techniques (in place of traditional FR-4 boards) clearly cuts overall environmental impacts, particularly for low-cost electronics.^109^ For example, a screen-printed radio frequency identification (RFID) sensor tag was shown to emit only ≈25–42 g CO_2_ over its life cycle, compared to ≈613 g CO_2_ for a similar device made by traditional PCB etching, over a 95% reduction in greenhouse gas (GHG) emissions.^110^

**Table 2:** Comparison of environmental impact of traditional CMOS and various printing technologies

Parameter	Traditional CMOS	Transfer printing	Contact printing	Direct roll printing	In-tandem transfer printing
Principle of operation	Lithography + etching (subtractive)	Interfacial adhesion between the structure or donor and stamp	Shear forces and van der Waals forces	van der Waals force	Shear force and van der Waals
Process type	Subtractive	Additive	Additive	Additive	Additive
Need for lithography	Multiple lithography steps and masks needed	Needed during the nanomaterial fabrication step	Needed for selective printing of nanomaterials	Needed during the nanomaterial fabrication step	Needed to make master template
Resolution	Nanometer scale	Micro- to nanometer	Micro-scale (donor limited)	Micro-scale (donor limited)	Donor limited
Tool cost & complexity	>$30 million, EUV tools; very high complexity	Cost of tool ≈ $100,000; low to medium complexity	Tool not yet commercialized; low complexity	Tool not yet commercialized; low to medium complexity	Tool not yet commercialized; low to medium complexity
Environmental impact	Heavy use of toxic chemicals & solvents	Less waste, fewer solvents	Low to moderate chemical use	Low to moderate chemical use	Low chemical use
Water consumption	High (e.g., ∼7 L/cm^2^ of ultrapure water/wafer)	Low (water mainly used in ink formulation)	Low (water mainly used in ink formulation)	Low (water mainly used in ink formulation)	Low (water mainly used in ink formulation)
Energy usage	High (high-temperature steps, CMP, etc.) (∼20–30 kWh per 12-inch wafer layer)	Low (∼0.05–0.5 kWh/m^2^)	Low (∼0.05–0.5 kWh/m^2^)	Low (∼0.5 kWh/m^2^)	Low (∼0.5–1 kWh/m^2^)
Hazardous waste	High (≈200 kg/wafer m^2^)	Minimal; <1 g/m^2^ (mostly dry, minimal chemicals)	Minimal; <1 g/m^2^ (mostly dry, minimal chemicals)	Minimal; <1 g/m^2^ (mostly dry, minimal chemicals)	Minimal; <1 g/m^2^ (mostly dry, minimal chemicals)
Contaminants	Acids, metals, PFOS, solvents	Minimal (organic solvents such as toluene, xylene, NMP, DMSO; surfactants such as Triton X-100, PEG)	Minimal (same as transfer printing)	Minimal	Minimal

EUV, extreme ultraviolet lithography; NMP, N-methylpyrrolidone.

These waste reductions are primarily attributed to the elimination of multi-step photolithography, acid etching, and extensive rinsing cycles, so that nearly all of the deposited ink remains in the final pattern instead of becoming waste.^106^ Consequently, by minimizing processing steps, printing methods accommodate the use of green, non-toxic inks. For example, printed thin-film transistors have even been demonstrated using water-based carbon nanotube inks, completely avoiding hazardous solvents and etchants.^111^ Although water-based printing still requires some rinsing, the total water consumption is far lower than the massive volumes of UPW (on the order of 10 million gallons per day) needed to clean and chemically process silicon wafers in a conventional fabrication facility.^112^ Accordingly, printed sensors and RFID circuits generate minimal chemical waste and produce negligible wastewater.^110^^,^^111^

Although printed electronics offer a promising route to reduce process complexity and minimize wastewater generation, they remain far from enabling complex ICs or large-scale integration.^4^ Most advanced systems are likely to remain hybrid, retaining silicon for high-performance logic and memory, while employing printed electronics for sensors, interconnects, or disposable components.^39^

Recent LCAs suggest that hybrid printed/silicon CMOS systems can reduce environmental burdens; for instance, optimizing a printed sensor tag with bio-based substrates and inks cut its printed-component global warming potential (GWP) by ∼39%.^110^ However, printable conductive inks, particularly those based on nanomaterials (e.g., silver nanoparticles), often require additives such as surfactants and dispersants to maintain colloidal stability, potentially introducing new waste streams and complicating purification processes.^113^^,^^114^ In contrast, additive printing technologies significantly reduce reliance on wet etching and high-volume UPW. The inherent trade-offs define the practical boundaries for printed electronics in achieving zero-discharge fabrication.

## Outlook

The semiconductor industry is going through massive advancements in digital, analog, tools, manufacturing technologies, as well as material domains. The chip development requires highly sophisticated and complex processes at all levels from design to production. Moving forward, the processes are going to need critical changes from architectural design to sustainable materials and end-to-end resource-efficient fabrication to address the growing demand for semiconductors. To achieve this, the industry needs to adopt the latest technologies that increase efficiency and production of highly advanced process nodes and lower the release of wastewater contaminants. With growth of semiconductor manufacturing, it will increasingly become difficult to ignore the ecological footprint.

Achieving close to zero discharge of contaminants will not only require cutting-edge technological innovation to detect and degrade complex toxic and hazardous contaminants released from the industry but also proactive policy interventions. Policymakers must acknowledge the “unknown” toxic and hazardous chemicals released and implement stringent regulations to increase transparency. This is essential, as accurately identifying these substances is the first step toward developing targeted and efficient treatment solutions. To balance transparency with competitiveness, the industry should adopt standardized confidential disclosure frameworks such as third-party audits or tiered disclosure systems that allow certain regulators and researchers to access chemical composition data to carry out the research under strict confidentiality agreements, while the public receives aggregated information. Furthermore, global standards could be made for chemical disclosure that protect proprietary information while ensuring safety and environmental compliance. In this regard, instead of revealing full chemical formulations and compositions, companies can disclose the amount, function, hazard class, or environmental impact of chemicals, which informs risk assessments without giving away trade secrets and allows designing proper treatment plans.

Although the above-mentioned solutions look promising, a significant gap remains in the form of fragmented regulatory landscapes. Currently, most policies addressing semiconductor environmental impacts are confined to national jurisdictions, while the industry itself is deeply globalized from products, materials, and waste crossing countries at every stage. This disconnect hampers coordinated efforts toward sustainability. As such, there is a pressing need for globally aligned policies that address the environmental footprint of semiconductor production comprehensively, from resource extraction to manufacturing to end-of-life disposal. Globalized supply chains require globalized environmental governance.

While current wastewater treatment technologies mitigate contaminants to some extent, a more sustainable and holistic approach lies in integrating resource-efficient approaches such as printed electronics with the conventional semiconductor fabrication processes. Advanced printing technologies offer a sustainable alternative to conventional semiconductor fabrication processes. Moreover, printed electronics can be applied to enhance monitoring and control systems. For instance, low-cost, flexible and disposable sensors could be produced for real-time tracking of the performance of wastewater treatment processes. Additionally, printed electronics could enable development of advanced, cost-effective filtration membranes based on nanomaterials, particularly for ultrafiltration, reverse osmosis, adsorption of toxic compounds, etc., thus further contributing to wastewater management. By reducing high-temperature steps and eliminating the need for hazardous chemicals, these methods significantly lower energy consumption and minimize environmental impact. As the industry continues to evolve, adopting these innovative approaches will be crucial for mitigating the environmental footprint of semiconductor manufacturing, paving the way for a more sustainable future. This transition not only offers a route to significantly reduce the volume of wastewater released but also offers new solutions for sustainable growth of semiconductor manufacturing sector.

The creation of more sustainable and circular pathways is both a challenging and exciting frontier for the global semiconductor industry. In the year 2023, the global economy was 7.2% circular, a decline from 9.1% in 2018, and was expected to worsen over the years.^115^ The current semiconductor fabrication processes are intensive in every aspect from material to water to energy, and their environmental impact is not only limited to the wastewater stream released from the manufacturing processes. There are considerable challenges associated with end-of-life disposal of electronics, which are leading to a tsunami of electronic waste (e-waste). Today, e-waste is the world’s single and fastest growing hazardous waste stream. Current semiconductor manufacturing relies on rare earth and toxic materials, extensively uses materials that hardly degrade under natural conditions, and requires high-energy-consuming manufacturing tools (e.g., extreme ultraviolet lithography and high-temperature processes).^116^

Reversing the predicted downward trend in circular approach will require reducing material extraction and consumption, making the processes additive rather than subtractive, enabling the design of renewable feedstocks and end-of-use considerations and transforming current materials to radically increase their sustainability. Different types of biodegradable materials are being investigated to develop transient electronic devices so that they lead to minimal e-waste at the end of life with zero to positive environmental impact. The semiconductor industry is a strategic sector for the green economy transition due to its role in enabling energy-efficient technologies, renewable energy systems, electric vehicles, smart grids, Internet of Things, and advanced manufacturing. By continuing to innovate and adopt eco-friendly practices, the semiconductor industry can contribute to a sustainable future. To be compliant with this mission, the semiconductor industry also needs to modify own processes. As an example, approximately 500 billion tons of materials have been consumed in the last 5 years, which is nearly equal to the consumption in the entire 20th century. There is considerable cost involved (anywhere from $10 to $100 million) to produce a single new material.^117^ At the same time, it must be noted that material design and sustainable material discovery take considerable time; it is not uncommon for it to take a decade or more. Here, advances in machine learning and autonomous experimentation could help.^118^^,^^119^ This approach can also be extended to the wastewater treatment coming from the semiconductor industry. For example, sludges resulting from semiconductor wastewater treatment can be reused in the productive cycle.^120^ Further, smart remediation system could be developed to selectively adsorb, transport, and deliver the anions and cations from the wastewater, which can be treated and reused. Such systems could operate autonomously in hazardous environments, offering precision cleanup while minimizing human exposure.

Nonetheless, it is challenging to balance the cost and performance while decarbonizing the semiconductor manufacturing processes. The development of new materials needs to achieve this balance, and a systems perspective that considers the full range of consequences of production, use, and reuse or recycling (also known as a cradle-to-cradle approach) can be helpful.^116^ Further, detailed and systematic post-degradation analysis and exploration into the toxicological implications of novel material complexes can bring advancement to the green and transient electronics to mitigate the environmental footprint.

## Acknowledgments

We thank the College of Engineering at 10.13039/100015257Northeastern University for support.

## Author contributions

Conceptualization, R.D.; literature survey, S.S., A. Z., V.V., Z.T., and R.D.; writing, S.S. and A.Z.; writing - review and editing, Z.T., V.V., and R.D.; supervision, R.D.

## Declaration of interests

The authors declare no competing interests.
